# Factors affecting Saudi medical students’ engagement during synchronous and asynchronous eLearning and their impacts on the students’ academic achievement: a national survey

**DOI:** 10.1186/s12909-024-05323-3

**Published:** 2024-03-29

**Authors:** Amal A. Alghamdi, Ghada F. Alyousif, Amani M. AlQarni, Fatma H. Amer, Taghreed O. Alfadhel, Rawan N. Almutairi, Shatha M. Almutairi, Anwar D. Almutairi, Nouf A. Hakami, Kholoud. Al Ghamdi

**Affiliations:** 1 Department of Family and Community Medicine, College of Medicine, Imam Abdulrahman bin Faisal University, Dammam, Saudi Arabia; 2 College of Medicine, Majmaah University, Al Majma’ah, Saudi Arabia; 3 College of Medicine, Qassim University, Alqassim, Saudi Arabia; 4 College of Medicine, Jazan University, Jazan, Saudi Arabia; 5 Department of Physiology, College of Medicine, Imam Abdulrahman bin Faisal University, Dammam, Saudi Arabia

**Keywords:** eLearning, Interaction, Barrier, Online lecture, Educational process, Medical student

## Abstract

**Background:**

Nowadays, especially after the COVID-19 pandemic, electronic learning (eLearning) has become a necessity in education. eLearning can be either synchronous, where classes are conducted in real-time, or asynchronous, where students can access the class material at any time. Student-instructor interaction has become essential to the educational process. In the literature, most studies have focused on the preferred methods of eLearning and the barriers to interaction in eLearning. Thus, this study aimed to investigate the factors that affect students’ interactions during eLearning and their impacts on students’ academic achievements.

**Methods:**

A national cross-sectional study was conducted among clinical and pre-clinical medical students who were attending universities in five regions of the Kingdom of Saudi Arabia. Data were collected using a bespoke online self-administered questionnaire covering sociodemographic features, eLearning barriers, preferences, and the impact of eLearning on students’ performance and understanding.

**Results:**

This study involved 1371 medical students, of whom 52.37% were male and 51.13% were in their pre-clinical years of medical college. Of the participants, 59.88% (*n* = 821) preferred synchronous modalities of eLearning, and 33.33% (*n* = 457) avoided interaction during synchronous lectures. The main predictors of avoiding interaction during online lectures were being male in the clinical years of medical studies, being in a quiet atmosphere, having difficulties using the eLearning platform, having a poor internet connection, having a visual learning style, being insecure, and the presence of opposite-sex students and facilitators. In addition, 12.25% students (*n* = 168) reported a lower grade point average (GPA), whereas 11.96% (*n* = 164) reported an improved GPA after eLearning compared with in-person/onsite learning sessions. The GPA fluctuation was related to gender, personality type, learning style, interaction, and eLearning modality preference. Moreover, the students’ understanding was enhanced by recorded lectures (*n* = 1,093, 79.72%) and supportive multimedia (*n* = 1,037, 75.64%), and the easy to use platform (*n* = 1037, 75.64%).

**Conclusion:**

The synchronous modality of eLearning was the preferred teaching method among the medical students. However, multiple individual, technical, and environmental factors affected their interaction, performance, and understanding during these sessions. Hence, future interventional research is recommended to overcome interaction barriers and enhance student performance and understanding of eLearning.

## Background

eLearning refers to the use of technology in the educational process [1]. Online lecture teaching modalities can be classified into synchronous and asynchronous [2]. Synchronous eLearning involves live broadcasting in the educational session and possibly live interactions, whereas asynchronous eLearning is when the academic session is recorded [2]. Synchronous eLearning simulates the traditional classroom by allowing teacher-student interaction, encouraging students to ask questions, and preventing students from feeling isolated [3].

The new advancement in technology and the production of various software programmes, especially video conference software, have facilitated the development of synchronous modalities of eLearning and encouraged student interaction [4]. Peer interaction is essential during the educational process; however, teacher-student interaction is considered the pillar of the educational process [5]. Each learning modality has its advantages in facilitating the teacher-student interaction. For example, the synchronous teaching method could be used for illustrating complex matters and supporting students while assigning them class work, whereas the asynchronous learning method can be used as a platform for instructors to provide feedback to students while reflecting on their performances or following up with them especially when a synchronous teaching modality cannot take place [3]. Today, the educational process has shifted from teacher-instructed learning to student-centred learning [6].

A previous study found that student-content interaction and engagement matters for the persistence of the educational process [7]. However, students’ motivation for eLearning and course engagement is the main challenge that might affect the continuity and success of eLearning [8]. In the literature, student engagement and interaction have been assessed using various methods such as evaluating interaction in discussions [9] and students’ clicking on the various activities in their learning platform [8]. It might be easier to assess student motivation and understand the barriers to engagement during online lectures with one-to-one or small-group mentoring [8].

During the COVID-19 pandemic restrictions, medical colleges in the Kingdom of Saudi Arabia shifted almost all their educational platforms, especially lectures, to online teaching from 2020 till the end of 2021. This shift might have affected medical students’ learning process and interaction and lowered their academic achievements. However, limited research has investigated interaction barriers and described the impacts of limited interaction and engagement on students’ academic achievements. In this study, we suggest that students’ interaction and engagement might be affected by internal factors, including students’ eLearning modality preferences, personality types, learning styles, and socio-demographic features. In addition, we assumed that motivation and engagement might be affected by external factors, including household, technical, and social factors. Thus, in this study, we aimed to identify which of these suggested factors might have influenced medical students’ eLearning engagement. We also aimed to understand the impact of students’ eLearning engagement on their academic performance as measured by their GPA fluctuation, exam performance, and subject understanding.

## Methods

This cross-sectional study covered the five regions of Saudi Arabia: North, South, West, East, and Middle. One study author from each region approached at least one university. Data were collected using a self-administered online questionnaire technique. This online data collection was chosen because of the COVID-19 pandemic restrictions, which made it difficult for the authors to approach participants for recruitment personally. For sampling, we first stratified the kingdom into various regions and approached data collectors from various universities within these regions. Data collectors approached students from the same university through a convenient sampling approach using the students’ WhatsApp accounts. This was done to expand the reach and accessibility of the questionnaires and obtain a representative sample while minimising the risk of using convenient sampling, as this approach would provide us with a balanced number of participants with various sociodemographic features, especially regarding sex, academic level, and universities. The data collectors were supervised by one of the authors to make sure they approached students as planned and encouraged them with frequent reminders to improve the response rate. The data collection process lasted 2 months, from July to August 2021. The minimum accepted sample size was estimated to be 341 using the Epi Info software (version 7.2.4.0), considering a 95% confidence interval, 5% margin of error, and 50% engagement rate of eLearning among the participants.

The used questionnaire was a bespoke English questionnaire, and its items were designed based on the literature and expert opinion. The questionnaire aimed to measure specific aspects of online teaching and interaction. Each item was used individually rather than creating a global score to assess the overall level of engagement. A small pilot study was conducted to examine its face validity and clarity before running this study. The collected data included sociodemographic features (sex, academic year, region, marital status, socioeconomic status, and grade point average (GPA) before and after enrolment in an online teaching modality), technology-related data (type of device, platform atmosphere, internet connection, internet-handling skills, and setting atmosphere), personal and social factors (personality, the feeling of safety during interactions with others, the feeling of being threatened by peers’ negative comments, and the presence of opposite-sex students), instructor-related factors (the instructor’s ability to answer immediately and judgment, and an instructor of the opposite sex). In addition, we included data related to subjective descriptions of the participants’ personality types (shy or outgoing) and their learning style (visual, audio, or sensory). Furthermore, data were collected to measure the avoidance of engagement in online lectures (yes or no), mode of teacher-student interaction (unidirectional or bidirectional), preference for eLearning teaching modality (synchronous or asynchronous), changes in accumulative GPA and online exam performance (decreased, increased, or the same), and the level of subject understanding (poor or good).

Ethical approval was received from the Imam Abdulrahman bin Faisal University (IAU) Institutional Review Board (IRB) committee (IRB-2023-01-051). In addition, all participants were informed regarding the purpose and methods of the study and voluntarily provided written consent before filling out the questionnaire. All methods were performed in accordance with the relevant guidelines and regulations of the IAU ethical board committee. Data were analysed using the STATA 17 software. The chi-square and Fisher exact tests were used to assess significant differences in the distribution of determinants between the study groups. Unadjusted and adjusted logistic regression models were used to estimate the odds of interaction avoidance. In addition, multinomial logistic regression models were used to estimate the relative risk ratio for a change in GPA before and after the implementation of the online lecture modality in medical teaching. The regression models that estimated the risk of GPA fluctuation were adjusted by including the two main epidemiological confounders that are gender and academic year. However, GPA was added later to the adjustment of the logistic models that estimated the risk of interaction avoidance.

## Results

The total number of participants was 1371 students from the included universities and colleges (Table 1). Most participants were from governmental universities (*n* = 976, 71.19%) and the central region (*n* = 50.91%). The distribution of the participants among the universities were as follows: Imam Abdulrahman bin Faisal University (*n* = 188, 18.18%), Qassim University (*n* = 149, 14.41%), Jazan University (*n* = 110, 10.64%), Unaizah College of Medicine (*n* = 97, 9.38%), Majmah University (*n* = 89, 8.61%), King Faisal University (*n* = 57, 5.51%), King Saud bin Abdulaziz University for Health Sciences (*n* = 28, 2.71%). Significant differences in eLearning and interaction methods were found among the universities in the different kingdom regions. In the absence of standardisation of teaching methods among the Saudi universities, the synchronous method of eLearning was the most commonly used (*n* = 862, 62.87%). However, recorded lectures were not always available amongst 50.40% (*n* = 691) of the included universities. The student interaction with the instructor during live lecture was bidirectional in 52.15% of the respondents (*n* = 467). After the lecture, the main method of interaction was via email (*n* = 554, 40.41%). Most participants were male (*n* = 718, 52.37%), senior-level students (*n* = 701, 51.13%), from the middle region of the Kingdom (*n* = 698, 50.91%), unmarried (*n* = 958, 69.88%), and of middle socioeconomic class (*n* = 990, 72.21%), and had a GPA of A or B (*n* = 1310, 95.55%; Table 2).

**Table 1:** Shows the distribution of the universities and teaching methods among the participants according to geographical region

		Saudi Arbia Regions														
		Western		Central		Eastern		Southern		Northern		Total		Chi-square test		
		N	%	N	%	N	%	N	%	N	%	N	%			
		111	8.10	698	50.91	313	22.83	127	9.26	122	8.90	1371	100	x	df	p
Number of universities	Government	4		8		2		3		4		21				
	Private	4		3		0		0		0		7				
Number of participants	Government	87	20.72	326	46.70	312	99.68	126	99.21	122	100.00	976	71.19	564.94	8	< 0.001
	Private	23	20.72	23	3.30	0	0.00	0	0.00	0	0.00	46	3.36			
	Missing	1	0.90	346	49.57	1	0.32	1	0.79	0	0.00	349	25.46			
Method of learning	Asynchronous	16	14.41	393	56.30	56	17.89	26	20.47	18	14.75	509	37.13	225.36	4	< 0.001
	Synchronous	95	85.59	305	43.70	257	82.11	101	79.53	104	85.25	862	62.87			
Recorded lectures made available	No	34	30.63	485	69.48	103	32.91	35	27.56	34	27.87	691	50.40	208.64	4	< 0.001
	Yes	77	69.37	213	30.52	210	67.09	92	72.44	88	72.13	680	49.60			
Interaction during live lecture	Asynchronous	16	14.41	393	56.30	56	17.89	26	20.47	18	14.75	509	37.13	255.49	12	< 0.001
	Bidirectional	56	50.45	160	22.92	154	49.20	50	39.37	47	38.52	467	34.06			
	Unidirectional	31	27.93	124	17.77	96	30.67	39	30.71	54	44.26	344	25.09			
	Missing	8	7.21	21	3.01	7	2.24	12	9.45	3	2.46	51	3.72			
Email interaction	Yes	44	39.64	194	27.79	201	64.22	71	55.91	44	36.07	554	40.41	133.46	4	< 0.001
	No	67	60.36	504	72.21	112	35.78	56	44.09	78	63.93	817	59.59			
Social media interaction	Yes	55	49.55	113	16.19	77	24.60	48	37.80	73	59.84	366	26.70	146.17	4	< 0.001
	No	56	50.45	585	83.81	236	75.40	79	62.20	49	40.16	1005	73.30			
Blackboard interaction	Yes	19	17.12	61	8.74	32	10.22	22	17.32	44	36.07	178	12.98	74.57	4	< 0.001
	No	92	82.88	637	91.26	281	89.78	105	82.68	78	63.93	1193	87.02			

**Table 2:** Shows a summary of the characteristics of the participants concerning their learning modality preference (*n* = 1371)

		eLearning Modality Preference					Chi-Square Test		
		Asynchronous		synchronous		Total	X^2^	df	P
		*N* = 550	%	*N* = 821	%	*N* = 1371			
Sex	Female	145	22.21	508	77.79	653	166.51	1	< 0.001
	Male	405	56.41	313	43.59	718			
Academic year	Preclinical	161	24.03	509	75.97	670	141.16	1	< 0.001
	Clinical	389	55.49	312	44,051	701			
Region	Western	60	54.05	51	45.95	111	86.22	4	< 0.001
	Middle	196	28.08	502	71.92	698			
	Eastern	166	53.04	147	46.96	313			
	North	67	52.76	60	47.24	127			
	South	61	50.00	61	50.00	122			
Marital status	Married	38	9.20	375	90.00	413	235.15	1	< 0.001
	unmarried	512	53.44	446	46.56	958			
Socioeconomic	Low	53	59.55	36	40.45	89	42.75	2	< 0.001
	Middle	345	34.85	645	65.15	990			
	High	152	52.05	140	47.95	292			
GPA	A	350	52.71	314	47.29	664			< 0.001*
	B	171	26.47	475	73.53	646			
	C	28	51.85	26	48.15	54			
	D	1	14.29	6	85.71	7			
Type Devices	Phone	47	49.47	48	50.53	95			< 0.001*
	Tablet	226	61.92	139	38.08	365			
	Laptops	228	55.21	185	44.79	413			
	PC	45	9.13	448	90.87	493			
	Others	4	80.00	1	20.00	5			
Learning style	Audio	108	61.36	68	38.64	176	386.95	3	< 0.001
	Visual	56	58.33	40	41.67	96			
	Sensory	328	63.69	187	36.31	515			
	Not sure	58	9.93	526	90.07	584			
Personality	Outgoing	306	31.38	669	68.62	975	107.14	1	< 0.001
	shy	244	61.62	152	38.38	396			

*p-value comes from Fisher’s exact test

The main reason for unsatisfaction with the eLearning experience was the inability to interact during the online lecture (*n* = 100, 33.33%), followed by the inability to download online materials (*n* = 47, 15.67%), the inability to open the website or program (*n* = 46, 15.33%), unclear font or text (*n* = 43, 14.33%), the inability to play multimedia materials on the platform (*n* = 38, 12.67%), and other unspecified reasons (*n* = 26, 8.67%). In addition, 37.86% of the participants used their universities’ Blackboard platform account to access online materials (*n* = 519). The remaining participants used live conference software such as Zoom and Microsoft Teams. Amongst the participants, 32.24% reported poor internet connection (*n* = 442), and only 7.88% reported poor internet skills (*n* = 186).

The internal factors that might have affected the participants’ engagement in relation to their preference for online teaching modalities are summarised in Table 2. Most participants preferred synchronous teaching (*n* = 821, 59.88%). Amongst the participants who preferred the asynchronous method, most were male (*n* = 405,73.64%), in their senior academic years (*n* = 389, 70.73%), unmarried (*n* = 512, 93.09%), and had an excellent GPA score (*n* = 350, 63.64%). The three main reasons mentioned for preferring asynchronous learning modalities were time flexibility (*n* = 438, 79.63%), the ability to replay the lecture to study it (*n* = 212, 38.54%), and the possibility of facing internet connection problems with synchronous online lectures (*n* = 60, 10.91%). Regarding technology, most participants (*n* = 1071, 78.12%) used their computers (*n* = 906, 66.08%) to access their online lessons, and almost three-quarters of them were satisfied with the devices they used.

Concerning the students’ online engagement and teacher-student interaction during synchronous teaching, around a quarter of the participants reported that the primary method of interaction during lectures was unidirectional (*n* = 350), which means that the facilitator was the one asking questions. On the other hand, approximately one-third of the participants reported that the primary method of interaction was bidirectional (*n* = 539), which means that both the students and facilitators asked and answered questions. Statistically significantly more males (*n* = 339,74.18%), senior students (*n* = 299, 65.43%), and students with a high GPA (*n* = 278, 60.83%; Table 3) preferred synchronous modalities but avoided interaction (*n* = 449, 98.25%).

**Table 3:** Shows a summary of the distribution of the factors that might have influenced the students’ engagement and interaction during online teaching (*n* = 1371)

		Engagement in Online Lecture Interaction					Chi-Square Test		
		Engage		Avoid engagement		Total	X^2^	df	P
		Number = 914	%	Number = 457	%	Number = 1371			
Sex	Female	535	81.93	118	18.07	653	130.71	1	< 0.001
	Male	379	52.79	339	47.21	718			
Academic year	Juniors	512	76.42	158	23.58	670	56.07		< 0.001
	Senior	402	57.35	299	42.65	701			
GPA	A	386	58.13	278	41.87	664			< 0.001*
	B	495	76.63	151	23.37	646			
	C	31	57.41	23	42.59	54			
	D	2	28.57	5	71.43	7			
Preference	Asynchronous	501	98.43	8	1.57	509	367.507	1	< 0.001
	synchronous	413	47.91	449	52.09	862			
Having quiet setting	Yes	773	71.38	310	28.62	1083	51.45	1	< 0.001
	No	141	48.96	147	51.04	288			
Good platform atmosphere	Yes	770	81.05	180	18.95	950	288.12	1	< 0.001
	No	144	34.20	277	65.80	421			
Poor internet connection	No	699	75.24	230	24.76	929	95.36	1	< 0.001
	Yes	215	48.64	227	51.36	442			
Limited internet skills	No	809	68.27	376	31.73	1185	10.10	1	0.001
	Yes	105	54.45	81	43.55	186			
Feel safe during interaction	Yes	836	73.33	304	26.67	1140	135.32	1	< 0.001
	No	78	33.77	153	66.23	231			
Device difficulties	No	814	68.52	374	31.48	1188	13.74	1	< 0.001
	Yes	100	54.64	83	45.36	183			
Instructor answer immediately	Yes	824	77.30	242	22.70	1066	243.73	1	< 0.001
	No	90	29.51	215	70.49	305			
The instructor judged me negatively	No	599	71.91	234	28.09	833	26.25	1	< 0.001
	Yes	315	58.55	223	41.45	538			
I feel threatened by negative comments	No	772	71.42	309	28.58	1081	51.86	1	< 0.001
	Yes	142	48.97	148	51.03	290			
Presence of opposite-gender students	No	774	70.43	325	29.57	1099	35.26	1	< 0.001
	Yes	140	51.47	132	48.53	272			
Opposite gender of the instructor	No	819	68.25	381	31.75	1200	10.85	1	0.001
	Yes	95	55.56	76	44.44	171			
Personality	Outgoing	707	72.51	268	27.49	975	51.92	1	< 0.001
	shy	207	52.27	189	47.73	396			
Learning style	Audio	98	55.68	78	44.32	176	293.4470	3	< 0.001
	Visual	37	38.54	59	61.46	96			
	Sensory	244	47.38	271	52.62	515			
	Not sure	535	91.61	49	8.39	584			

*p-value comes from Fisher’s exact test

Concerning the estimated risk of avoiding interaction during online lectures, the odds of avoiding was at least two times higher amongst the participants who reported unsatisfaction with the provided platform atmosphere (adjusted OR = 6.27, 95% confidence interval [CI] = 4.79–8.21), the instructor’s slow response (adjusted OR = 6.84, 95% CI = 5.09–9.20), not feeling safe during interaction (adjusted OR = 4.41, 95% CI = 3.21–6.04), the presence of opposite-sex students during online lectures (adjusted OR = 2.05, 95% CI = 1.55–2.71), poor internet connection (adjusted OR = 2.24, 95% CI = 1.74–2.89), and the feeling of being threatened by other students’ negative comments (adjusted OR = 2.05, 95% CI = 1.55–2.71; for further details, please refer to Table 4).

**Table 4:** Shows a summary of logistic regression models for estimating the odds of avoiding engagement and online interaction among the study participants (*n* = 1371)

Variables	Categories	The odds of avoiding engagement in online lecture interaction			
		Un adjusted OR	95% CI	Adjusted OR	95% CI
Sex*	Female	**0.25**	**0.19 to 0.31**	**0.29**	**0.22 to 0.37**
	Male	Ref.		Ref.	
Academic year**	Preclinical	Ref.			
	Clinical	**2.41**	**1.91 to 3.04**	**1.60**	**1.24 to 4.48**
GPA***	A	Ref.		Ref.	
	B	**0.42**	**0.33 to 0.54**	**0.64**	**0.49 to 0.82**
	C	1.03	0.59 to 1.81	0.76	0.43 to 1.35
	D	3.47	0.67 to 18.02	4.56	0.81 to 25.69
Preference****	synchronous	Ref.			
	Asynchronous	0.01	0.01 to 0.02	0.01	0.00 to 0.02
Having quiet sitting ****	Yes	Ref.			
	No	**2.60**	**1.99 to 3.39**	**1.93**	**1.46 to 2.56**
Unsatisfied by the platform atmosphere****	Yes	Ref			
	No	**8.23**	**6.35 to 10.66**	**6.27**	**4.79 to 8.21**
Poor internet connection****	No	Ref			
	Yes	**3.21**	**2.53 to 4.07**	**2.24**	**1.74 to 2.89**
Limited internet skills ****	No	Ref.			
	Yes	**1.66**	**1.21 to 2.27**	1.15	0.82 to 1.59
Feel safe during interaction****	Yes	Ref		Ref.	
	No	**5.39**	**3.99 to 7.30**	**4.41**	**3.21 to 6.04**
Device difficulties****	No	Ref			
	Yes	**1.24**	**1.01 to 1.53**	1.20	0.86 to 1.67
Instructor answers immediately****	Yes	Ref.			
	No	**8.13**	**6.12 to 10.81**	6.84	5.09 to 9.20
Instructor judging me****	No	Ref.			
	Yes	**1.81**	**1.44 to 2.28**	0.93	0.71 to 1.21
I feel threatened by negative comments****	No	Ref			
	Yes	**2.60**	**1.99 to 3.95**	**2.05**	**1.55 to 2.71**
Presence of opposite-gender students****	No	Ref			
	Yes	**2.25**	**1.71 to 2.94**	**2.64**	**1.94 to 3.59**
Opposite gender of instructor ****	No	Ref.			
	Yes	**1.72**	**1.24 to 2.38**	**1.52**	**1.08 to 2.14**
Personality****	Outgoing	Ref.			
	shy	**2.41**	**1.89 to 3.07**	**1.48**	**1.14 to 1.92**
Learning style ****	Audio	Ref.			
	Visual	**2.00**	**1.21 to 3.33**	**2.00**	**1.20 to 3.35**
	Sensory	1.39	0.99 to 1.96	1.36	0.96 to 1.93
	Not sure	0.12	0.08 to 0.17	**0.14**	**0.09 to 0.23**

*Adjusted for academic year, **adjusted for sex, ***adjusted for sex and academic year, and ****adjusted for sex, academic year, and GPA. The bold font indicates a significant p value (< 0.05)

**Fig. 1:**
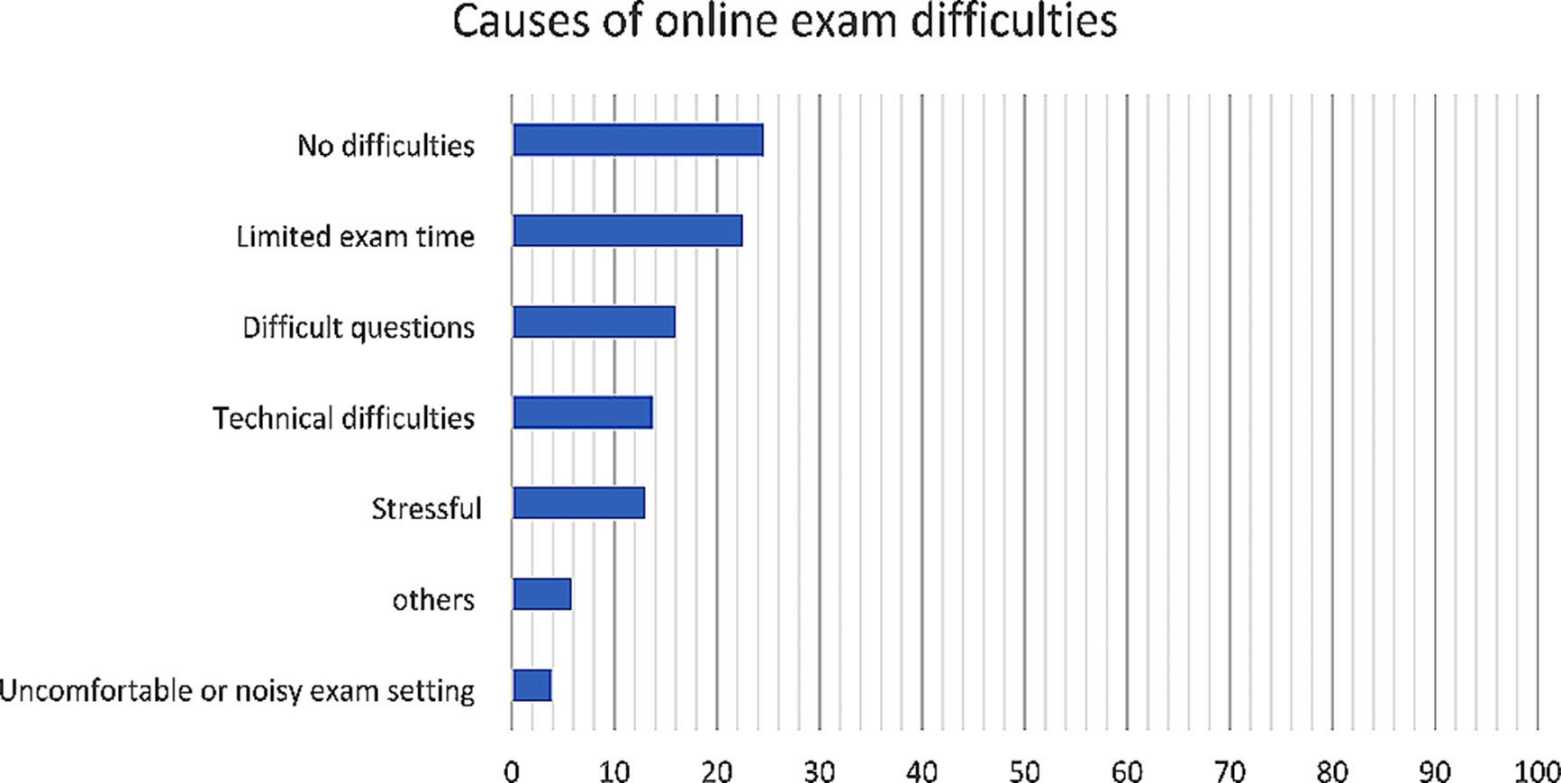
Shows the distribution of the factors that influence the difficulty of taking an online exam

Regarding exam performance and subject understanding, almost half (*n* = 477, 54.33%) of the participants were generally satisfied with their online exam performance. In the comparisons of onsite teaching and assessment scores, a small percentage reported lower and higher GPAscompared to their scores before the online lectures’ modality, (12.25% and 11.96%, respectively). Figure 1 shows that the major reported difficulty concerning the online exams was the limited allocated time for each exam (*n* = 308, 22.47%). On the other hand, few participants (*n* = 53, 3.87%) reported that the source of difficulty was the noisy or uncomfortable setting during the online exam. Concerning the improvement of the students’ understanding of the online learning process, 79.72% (*n* = 1,093) of the students reported that the recorded lecture material enhanced their understanding of the subject. Moreover, three-quarters (*n* = 1,037, 75.64%) of the participants found that the use of multimedia (e.g. educational videos, animated pictures, and audios clips) enhanced their understanding and that the type of platform used played an essential role in improving their understanding of the subject. Regarding the participants’ performances and effect of online teaching, 12.25% of the participants reported a decrease and 11.96% reported an improvement in accumulative GPA. The odds of the decline in GPA was greater amongst the senior students (adjusted OR = 3.25, 95% CI = 2.14–4.96), the participants with a GPA of B (adjusted OR = 2.08, 95% CI = 1.42–3.05), and the participants with a shy personality (adjusted OR = 1.50, 95% CI = 1.03–2.18). On the other hand, the odds of improving the accumulative GPA was associated with a preference for asynchronous teaching (adjusted OR = 1.55, 95% CI = 1.08–2.24) and avoidance of engagement in online interaction (adjusted OR = 1.55, 95% CI = 1.06–2.17; Tables 5 and 6).

**Table 5:** Shows a summary of the distribution of the factors that might have influenced the fluctuation in student GPA during the shift to online learning among the study participants (*n* = 1371)

		No difference		Lower GPA		Improved GPA		Total	Chi-Square Test		
		*N* = 1039	75.78%	*N* = 168	12.25%	*N* = 164	11.96%	*N* = 1371	X^2^	df	P
Gender	Female	563	86.22	40	6.13	50	7.66	653	75.44	2	< 0.001
	Male	476	66.30	128	17.83	114	15.88	718			
Academic year	Juniors	546	81.49	33	4.93	91	13.58	670	65.94	2	< 0.001
	Senior	493	70.33	135	19.26	73	10.41	701			
GPA	A	493	74.25	56	8.43	115	17.32	664			< 0.001
	B	522	80.80	78	12.07	46	7.12	646			
	C	20	37.04	31	57.41	3	5.56	54			
	D	4	54.14	3	42.86	0	0.00	7			
Region	Western	76	68.47	19	17.12	16	14.41	111	70.98	8	< 0.001
	Middle	573	82.09	68	9.74	57	8.17	698			
	Eastern	236	75.40	37	11.82	40	12.78	313			
	North	83	65.35	11	8.66	33	25.98	127			
	South	71	58.20	33	27.05	18	14.75	122			
Marital status	Married	390	94.43	9	2.18	14	3.39	413	112.38	2	< 0.001
	Unmarried	649	67.75	159	16.60	150	15.66	958			
Socioeconomic	Low	55	61.80	18	20.22	16	17.98	89	22.51	4	< 0.001
	Middle	778	78.59	99	10.00	113	11.41	990			
	High	206	70.55	51	17.47	35	11.99	292			
Preference	synchronous	663	80.76	82	9.99	76	9.29	821	27.77	2	< 0.001
	Asynchronous	376	68.36	86	15.64	88	16.00	550			
Interaction	Avoid	723	79.10	104	11.38	87	9.52	914	19.39	2	< 0.001
	Engage	316	69.15	64	14.00	77	16.85	457			

**Table 6:** Shows the summary of the multinomial logistic models for estimating the odds of fluctuation in GPA during the shift to online learning among the students (*n* = 1371)

		Un adjusted models						Adjusted models					
		No difference		Lower GPA		Improved GPA		No difference		Lower GPA		Improved GPA	
		OR	95%CI	OR	95% CI	OR	95% CI	OR	95% CI	OR	95% CI	OR	95% CI
Gender*	Female			**0.26**	**0.18, 0.38**	**0.37**	**0.26,0.53**			**0.39**	**0.26, 0.59**	**0.31**	**0.21, 0.45**
	Male	**Ref**						**Ref**					
Academic year	Preclinical	Ref						Ref					
	Clinical			**4.53**	**3.04, 6.76**	0.89	0.64, 1.23			**3.25**	**2.14, 4.96**	**0.59**	**0.41, 0.84**
GPA	A	Ref.						Ref.					
	B			1.32	0.91,1.89	**0.38**	**0.26, 0.54**			**2.08**	**1.42, 3.05**	**10.34**	**5.61, 19.15**
	C or D			**12.47**	**6.91, 22.52**	0.54	0.16, 1.81			**0.48**	**0.32, 0.72**	0.53	0.16, 1.81
Marital status	Married			0.73	0.35, 1.52	1.21	0.65, 2.25			0.64	0.30, 1.38	1.59	0.83, 3.01
	Unmarried	Ref.						Ref.					
Socioeconomic	Low			1.32	0.72, 2.44	1.71	0.88, 3.32			1.48	0.79, 2.80	1.61	0.87, 2.96
	Middle	Ref.						Ref.					
	High			**0.51**	**0.35, 0.74**	0.85	0.57, 1.29			**1.72**	**1.14, 2.59**	0.92	0.60, 1.41
Preference	synchronous	Ref.						Ref.					
	Asynchronous			**1.85**	**1.33, 2.57**	**2.04**	**1.46, 2.57**			1.16	0.80, 1.68	**1.55**	**1.08, 2.24**
Interaction	Avoid			**1.41**	**1.00, 1.97**	**2.02**	**1.45, 2.83**			0.87	0.59, 1.26	**1.52**	**1.06, 2.17**
	Engage	Ref						Ref					
Personality	Outgoing	Ref.						Ref.					
	shy			**2.22**	**1.59, 3.11**	**1.49**	**1.05, 2.13**			**1.50**	**1.03, 2.18**	1.03	0.71, 1.51
Learning style	Audio			Ref.									
	Visual			1.29	0.68, 2.43	1.11	0.57, 2.18			1.03	0.51, 2.06	1.05	0.53, 2.10
	Sensory			0.74	0.47, 1.18	0.77	0.48, 1.24			0.69	0.42, 1.13	0.72	0.44, 1.17
	Not sure			**0.28**	**0.17, 0.46**	**0.33**	**0.20, 0.54**			**0.52**	**0.29, 0.93**	**0.41**	**0.23, 0.73**

*Adjusted for the academic year, **adjusted for sex, and ***adjusted for the academic year and sex. The bold font indicates a significant p value (< 0.05)

.

## Discussion

The main aim of our study was to identify factors that influence student interaction and academic achievement when using eLearning modalities. This study shows that more than half of the medical students preferred the synchronous modality of online teaching, and almost one-third did not engage in the class, most of whom were senior male students. Khalil et al. (2020) recently reported that Saudi medical students were satisfied with the synchronous eLearning modality during the COVID-19 pandemic and that there is an urgent need to shift to eLearning [2]. However, the main reasons that some of our participants preferred recorded lectures during online teaching were the time flexibility, ability to replay the lessons, and possibility of internet problems interfering with synchronous online instruction and sessions. These reasons highlight the importance of the use of cutting-edge technologies in online education and the need for offline teaching materials to refer to after the online sessions. In addition, the challenges in the use of technology in eLearning are frequently encountered in urban areas where limited access to the internet might affect the synchronisation process of eLearning [10]. Hence, demand is higher for providing asynchronous eLearning modality as a solution for students who might have problems accessing their synchronous classes. On the contrary, many studies have claimed that asynchronous education was superior to synchronous education in terms of flexibility in time and place, higher student independence, and self-monitoring [11–13]. However, these findings were poorly studied owing to methodological limitations such as unconsidered confounders and the inconsistency in the used technologies [11].

In addition, one main factor reported in the literature regarding successful eLearning implementation was related to students’ efficacy and belief in their abilities to practice eLearning, as these motivate them to learn and develop their technical skills [12]. Conversely, the main barriers reported were related to administrative issues such as complexity and cost- and payment-related issues [1]. Furthermore, difficulty in using the platform and difficulty of interaction due to the platform atmosphere were also critical factors for users to refrain from using online services, especially when they could shift to another provider [1]. In addition, technical issues such as internet problems and unequal online learning opportunities were considered the major contributing factors to complicated online learning [14]. Nevertheless, other eLearning barriers related to teachers were reported, including a lack of knowledge of the online teaching environment, the facilitator’s lack of skills, and the difficulty in assessment and evaluation [1, 15]. Furthermore, the other reported eLearning barriers were related to the given curriculum, including ambiguity, quality of resources, assessment methods, teaching process, and the colleges themselves, such as organisational and physical structural factors not aligned with online learning and the lack of adequate resources [15]. Regarding these barriers, the students showed interest in eLearning when the eLearning process was well structured, of high quality, and supported by blended traditional learning and tutorials [16]. Baticulon et al. (2021) reported in their national survey in the Philippines that only 41% of students engaged mentally and physically in online learning activities during the COVID-19 pandemic. The most frequently reported barriers were poor communication with facilitators, the need for more facilitator direction, and home responsibilities [17]. The lack of student interaction and engagement and effective facilitator-student communication might be considered the main barriers to transition from a physical to an online environment; thus, careful consideration should be given to solve this matter [18].

In our study, most participants who avoided interaction were male, were at the senior level, and had a higher GPA. In the literature, the risk of avoiding interaction was higher in the following student categories: those who were not satisfied with their teaching platform, those who had slow-responding instructors, those who had mixed male and female sessions, those with a poor internet connection, and those who felt threatened by other students’ comments. Moreover, personal characteristics were found to play a role in students’ engagement during online teaching [19]. Emotional factors such as anxiety and enjoyment are essential in determining student engagement [11]. In addition, gender directly influences the educational learning process, especially in cultures with gender boundaries [12]. Similarly, the role of trust and privacy among learners might enhance or limit students’ interactions in their classes [1]. Learning style also played a role in avoiding interaction. The students in this study who described their learning style as visual showed a higher risk of avoiding interaction during online sessions. The literature recommends that online courses should be designed to accommodate the various learning styles and needs of students to enhance their engagement and academic achievement [20]. A previous study suggested that with the advancement in information and communication technology, eLearning strategies could be enhanced to become ‘adaptive’ to learners’ varied learning needs [21]. Many researchers have implemented artificial intelligence to recognise students’ learning styles and create ‘adaptive’ eLearning platforms and resources. Most of our students interacted during their sessions using a unidirectional method. A Bidirectional teacher-student interactions are well known to strengthen the role of student engagement during online sessions and improve student understanding by asking questions. The main contents of teacher-student interactions were asking about illustrations or content, planning work tasks and organising responsibilities, and providing emotional support and guidance [3]. However, online teaching could limit student interaction, as most students reported little interaction with their instructors [20]. However, technological advancements, especially faster internet speed and video conference software, might enhance student interaction [4].

Our study assessed the effect of student interaction and engagement in various eLearning modalities by assessing exam performance satisfaction, GPA fluctuations, and subject understanding. Satisfaction with online exam performance might reflect the success of the eLearning process, especially in the absence of other interfering environmental or technical factors. Approximately half of our students were satisfied with their exam performance. The main reasons for dissatisfaction were the limited allocated time, challenging questions, technical difficulties, stress, and uncomfortable or noisy online exam setting. Khalaf et al. (2020) reported that online exam satisfaction mainly depends on the availability of technology that is easy for students to use and allows them to navigate exam questions without difficulties or interruption. They also reported that online exam timing should consider the variation in technological skills amongst students (e.g. typing skills), internet speed and quality, revision time, and question backtracking [21]. However, designing online exams to accommodate these factors should not influence situations that could facilitate cheating. The literature has identified the presence of an ‘opportunity to cheat’, which could result from the design and delivery of the exam, as one of the most important reasons to cheat during the online exam. Interventions such as remote proctoring, introducing codes of conduct during examinations to the students before the exam, substituting individual assignments for group assignments, and using open-book exams could be adapted to reduce the incidence of cheating [13].

Student satisfaction with their eLearning was an essential factor of learning persistence, which pertains to students’ enthusiasm to accomplish their learning goals and overcome learning difficulties and challenges [7]. One factor that enhanced student satisfaction, motivation, and course score was technology self-efficacy [22]. This suggests the need to improve students’ technological skills while simultaneously enhancing their learning skills. In our study, the students believed that synchronous lectures enhanced their understanding mainly when multimedia (e.g. educational videos, animated pictures, and audios clips) and uncomplicated platforms were used for their eLearning. A systematic review of randomised control trials that included 6750 participants found that knowledge gained through eLearning did not differ from knowledge gained through traditional learning. On some occasions, eLearning was superior to traditional education, especially when eLearning was blended with traditional learning [23]. The study also reported that different eLearning modalities did not affect the amount of knowledge gained [23]. One main challenge in obtaining eLearning educational outcomes is that these are mainly learner dependent, influenced by students’ commitment, self-motivation, and self-monitoring [13]. Students’ loyalty to eLearning statistically significantly correlated with the overall eLearning service quality [24]. In our study, the risk of decreased GPA was higher amongst the senior students, students with an average GPA, and students with shy personalities. On the other hand, the GPA improved in the students who preferred asynchronous teaching. This might be attributed to the fact that asynchronous modalities allowed participants to replay recorded lectures and refer to educational materials as needed at their own appropriate time, giving them more flexibility. In addition, the participants described themselves as shy and were worried about their peers’ and instructors’ responses if they interacted during synchronous lessons. Hence, individualised eLearning educational plans should be recommended depending on the student’s demands and needs. eLearning outcomes significantly moderately correlated with the course design and the teacher-student interaction [25], illustrating the need for better online course designs and considering the students’ personalities.

## Strengths and limitations

A significant strength of this study is that it is a multi-university study with participants from all regions of Saudi Arabia. It also highlights students’ perceptions regarding their preferences for eLearning modalities and the barriers to their engagement in such modalities. One limitation of this study is its use of online survey methods, which might have subjected the data to bias. However, we made a great effort to ensure that students with various sociodemographic and academic characteristics had sufficient access to the questionnaires. We used a systematic questionnaire distribution and asked data collectors from each university to distribute the questionnaire. Another limitation of this study is the use of a cross-sectional design, which might have limited the understanding of the risk factors for poor interaction and performance due to the lack of temporal sequences and the risk of unadjusted or latent unmeasured confounders. Furthermore, the use of unvalidated questionnaire that measured retrospective events might have led to measurement errors due to recall bias, especially considering the absence of a proper validation study. Finally, the subjective assessment of some study variables such as GPA, level of interaction, and exam performance might have led to measurement errors and recall biases being inherited into the study estimates.

## Conclusion

This study sheds light on students’ perceptions of their engagement during online educational process. As part of their engagement and teacher interaction, a large percentage of the students preferred synchronous online lecture modalities. However, students who preferred the asynchronous teaching modality showed better GPAs and were more satisfied with their online exam performance. This reflects that the students’ engagement and academic benefit from eLearning do not solely depend on synchronous teacher-student interactions. Other factors were identified to affect students’ engagement during their eLearning process, including having an outgoing personality, learning style, and the availability of fundamental technology (e.g. diverse multimedia, good internet connection, and easy interactive platforms). Finally, both synchronous and asynchronous eLearning are a promising tool in education and complement each other to achieve better academic performance. Keeping students engaged and motivated to attend such online classes is a challenge, but success in keeping them engaged affects their academic achievement and satisfaction.

## Data Availability

The datasets generated and/or analysed during the present study are not publicly available owing to the ethical confidentiality agreement but are available from the corresponding author upon reasonable request.
